# Supplementary figures and images for: Genome-wide transcription response of Staphylococcus epidermidis to heat shock and medically relevant glucose levels
Source: Front Microbiol. 2024 Jul 22;15:1408796. doi: 10.3389/fmicb.2024.1408796 (PMC11298487; doi:10.3389/fmicb.2024.1408796)

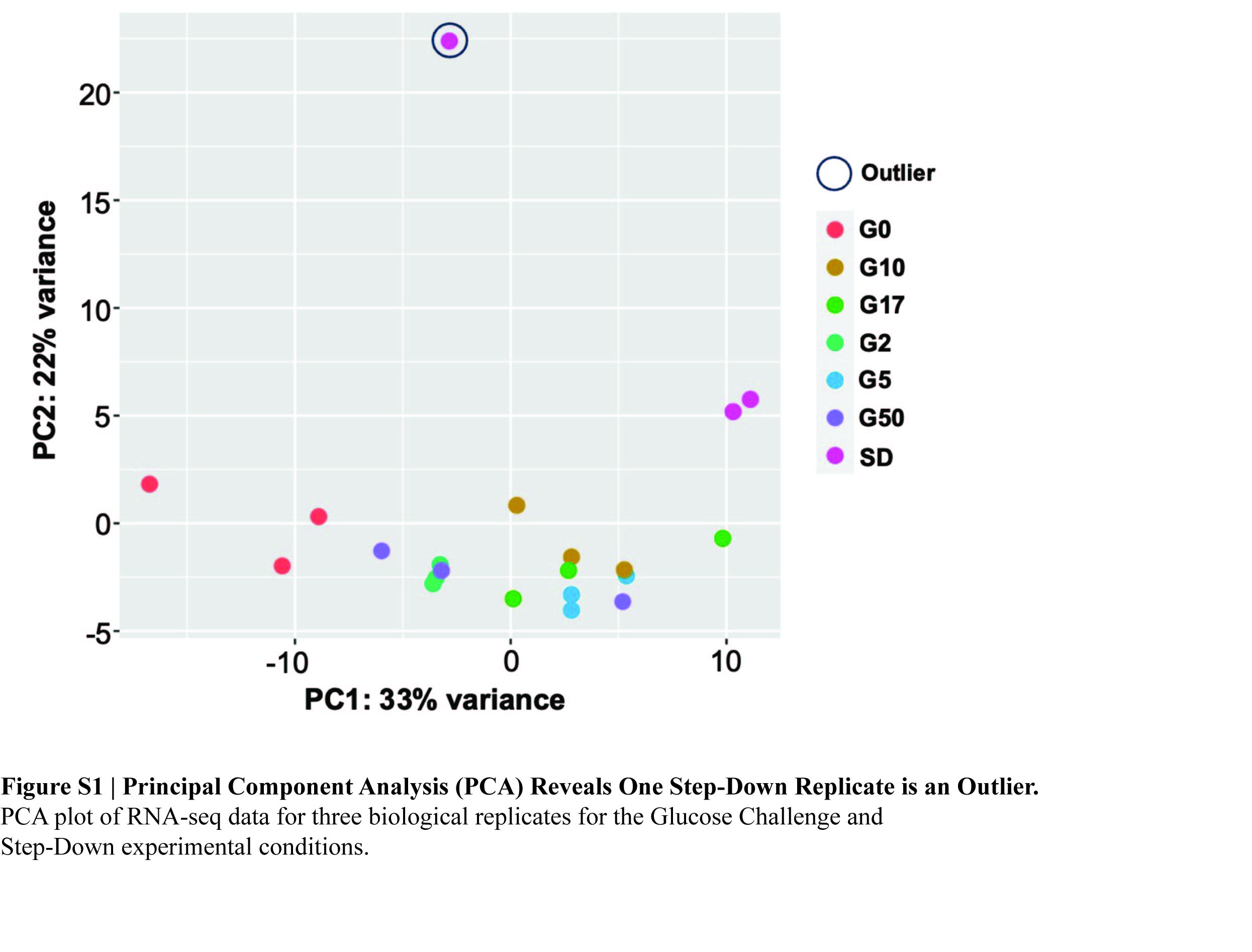

Supplement: Supplementary file 1 [file Image_1.JPEG]

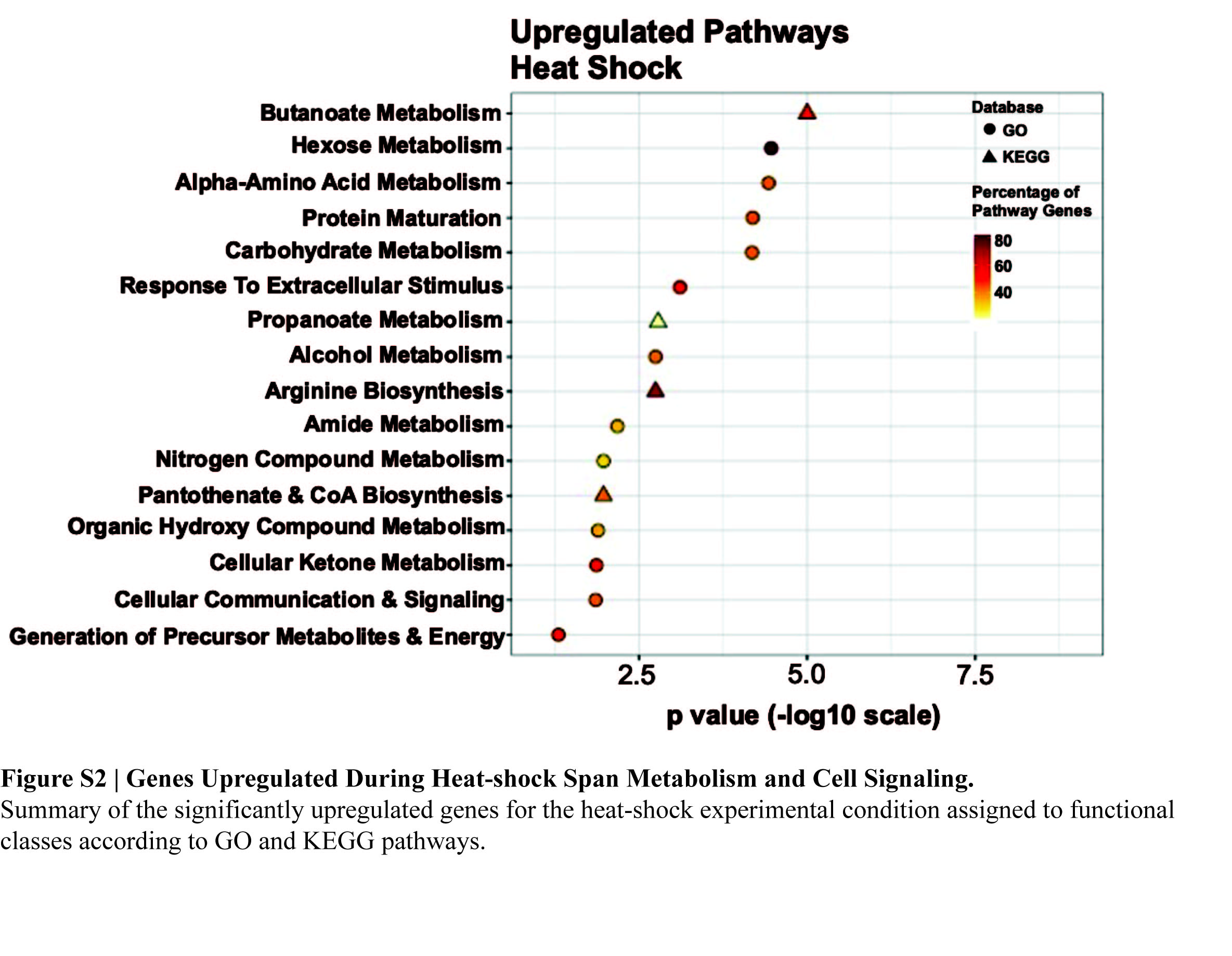

Supplement: Supplementary file 2 [file Image_2.JPEG]

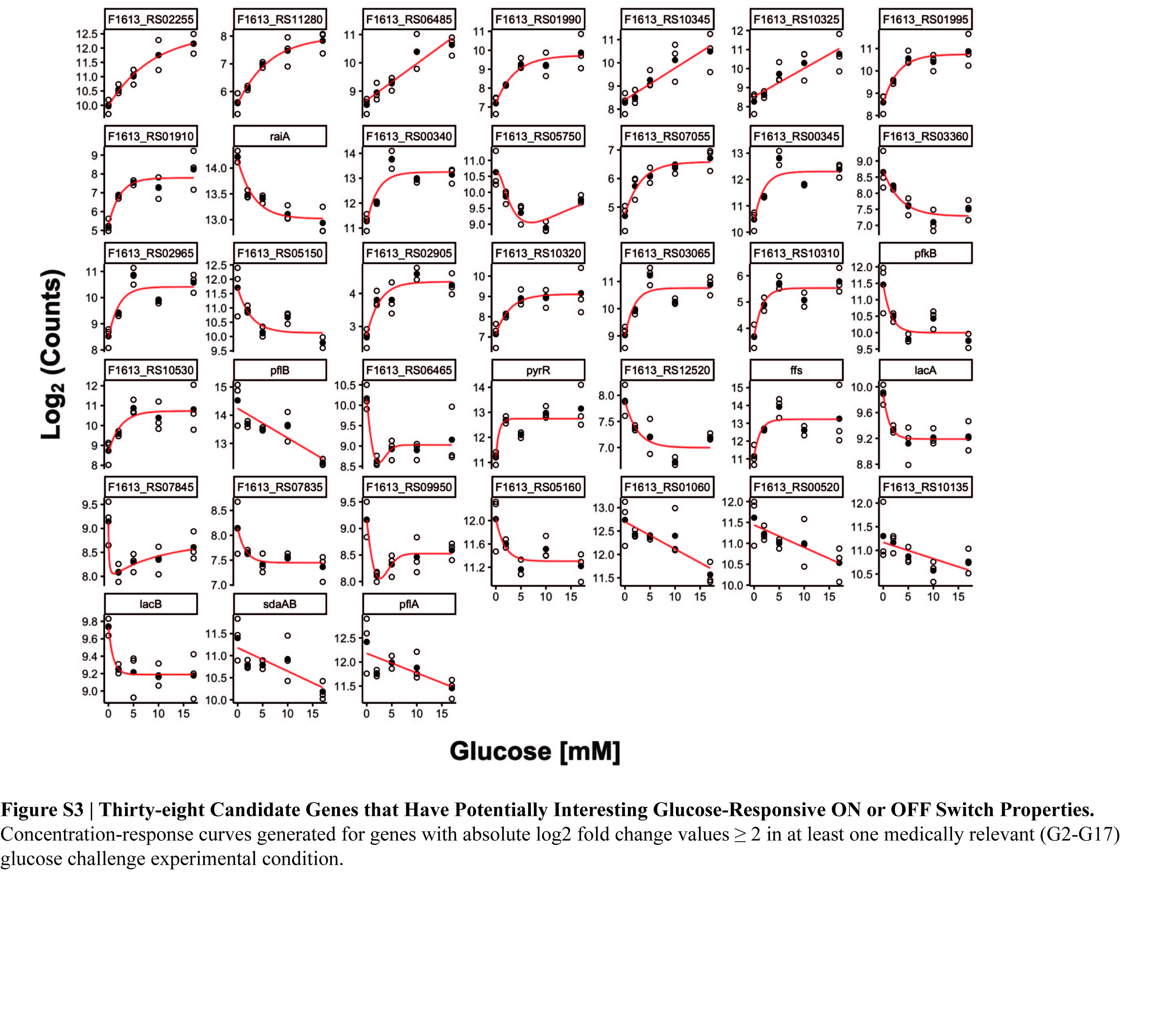

Supplement: Supplementary file 3 [file Image_3.JPEG]

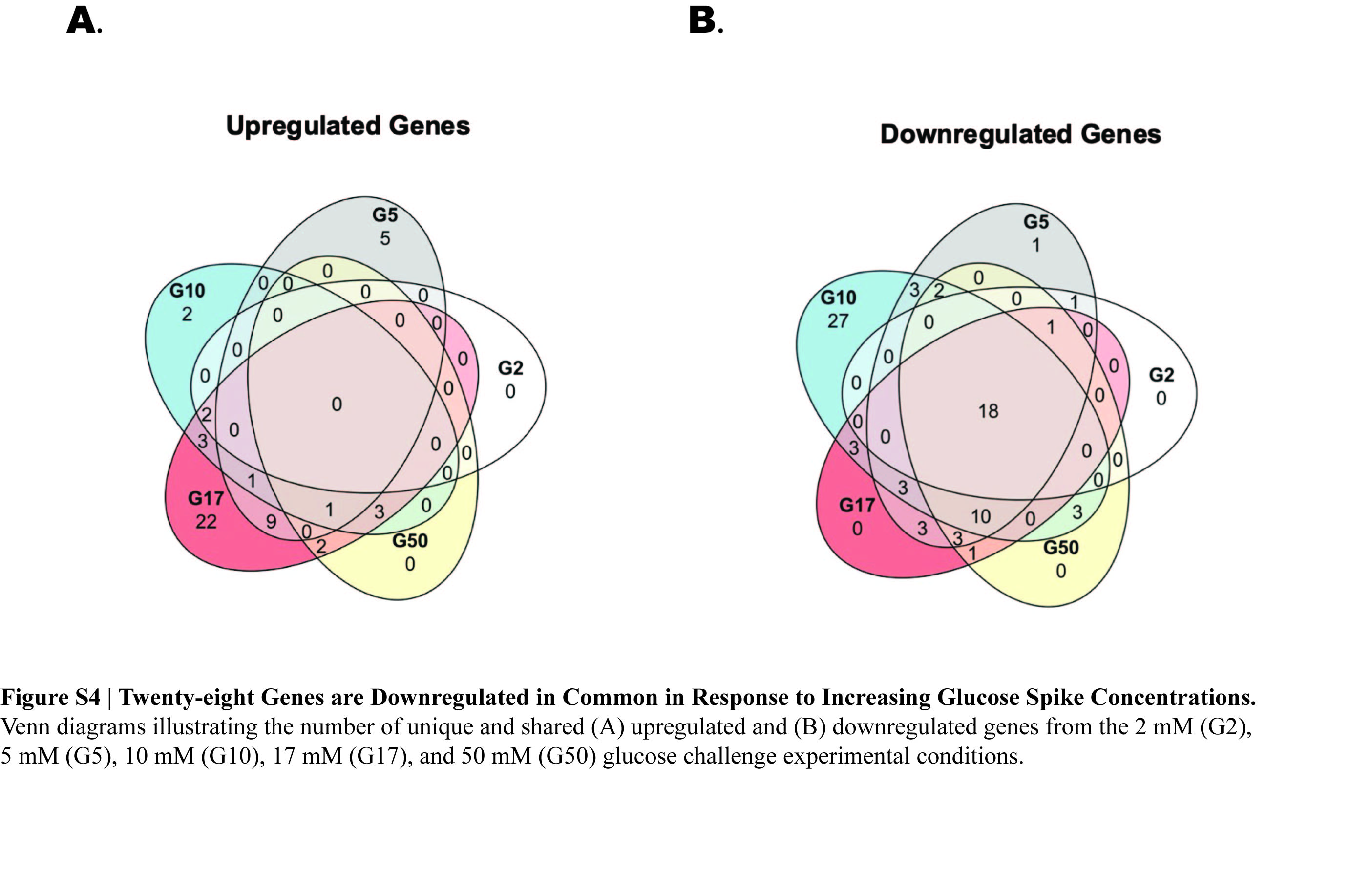

Supplement: Supplementary file 4 [file Image_4.JPEG]

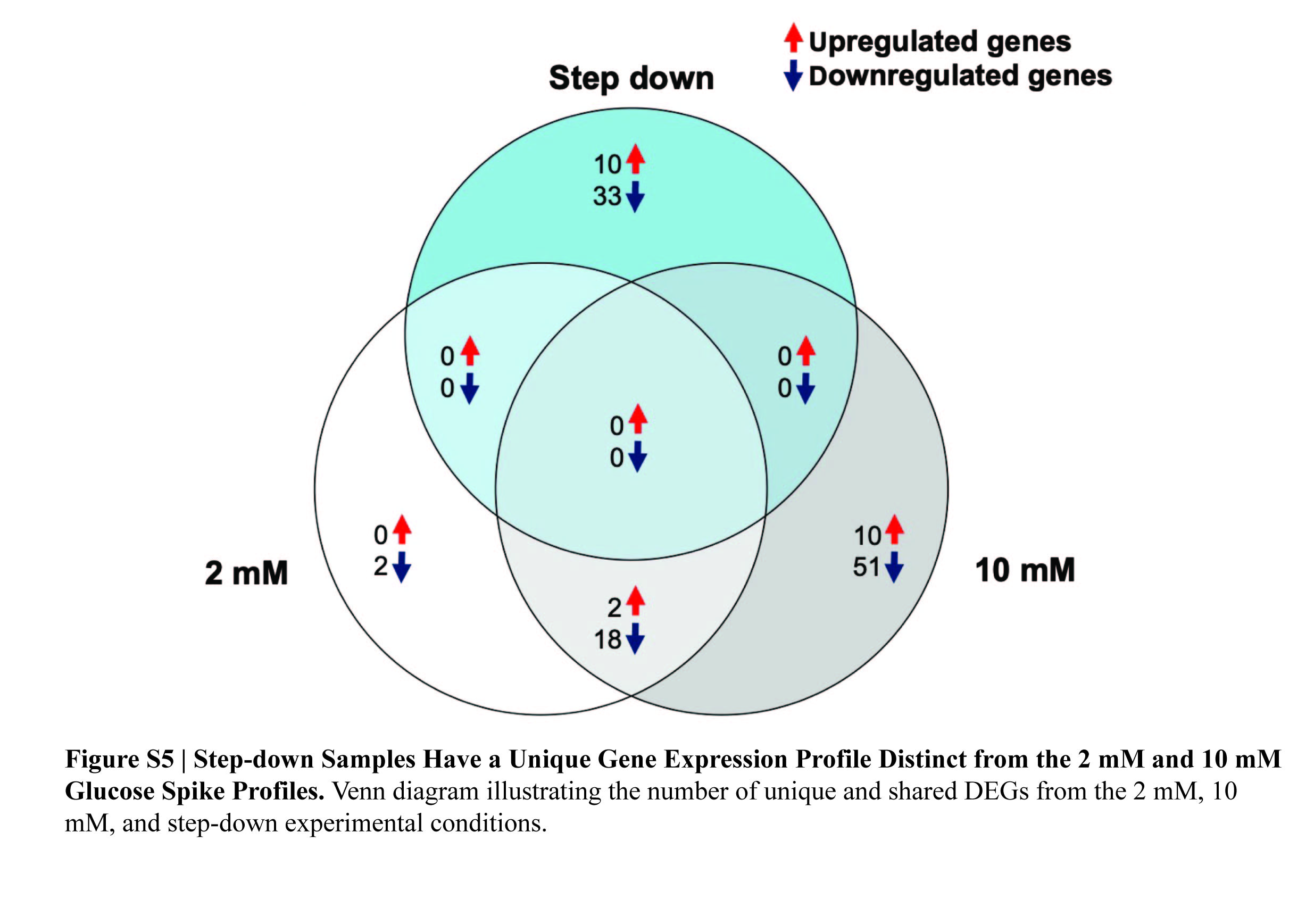

Supplement: Supplementary file 5 [file Image_5.JPEG]

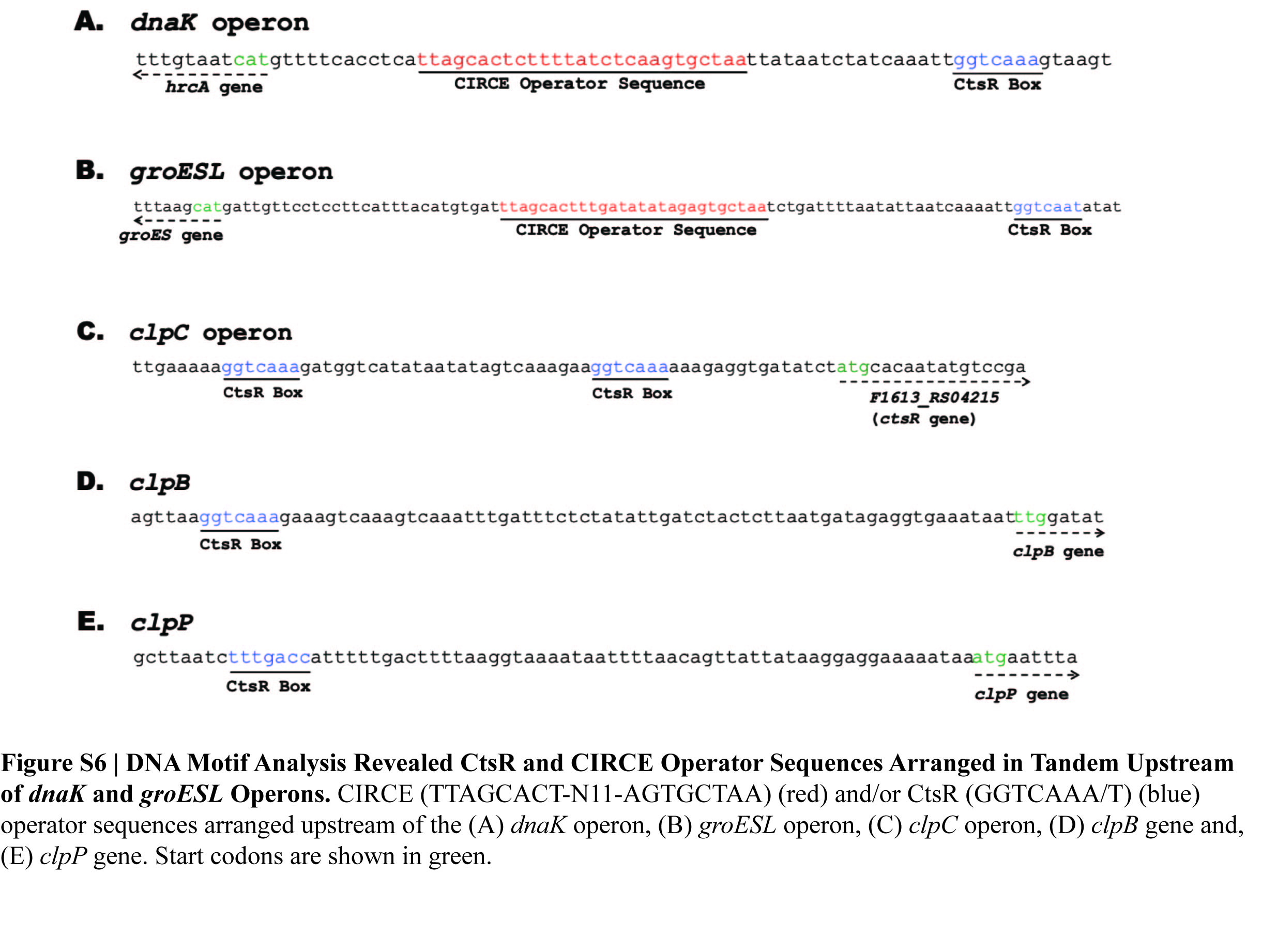

Supplement: Supplementary file 6 [file Image_6.JPEG]
