# Supplementary material for: Genome-wide transcription response of Staphylococcus epidermidis to heat shock and medically relevant glucose levels
Source: Front Microbiol. 2024 Jul 22;15:1408796. doi: 10.3389/fmicb.2024.1408796 (PMC11298487; doi:10.3389/fmicb.2024.1408796)
Supplement: Supplementary file 7 [file Table_1.DOCX]

**Table S1 | S. epidermidis heat-shock induced transcripts.**

| **Functional Class**  **& Symbol** | **Locus** | **Name** | **log2FC** | **Adjusted p-value** |
| --- | --- | --- | --- | --- |
| **Detoxification** |  |  |  |  |
|  | F1613_RS03775 | flavocytochrome c | 3.92 | 5.04E-15 |
|  | F1613_RS06270 | truncated hemoglobin YjbI | 1.90 | 1.52E-06 |
|  | F1613_RS09765 | SACOL1771 family peroxiredoxin | 1.87 | 8.88E-08 |
|  | F1613_RS04585 | heme-dependent peroxidase | 1.73 | 5.74E-07 |
|  | F1613_RS03465 | DsrE/DsrF/DrsH-like family protein | 1.60 | 5.79E-03 |
| **DNA Integration** |  |  |  |  |
|  | F1613_RS12560 | Integrase | 1.74 | 6.66E-03 |
| **DNA/RNA Repair** |  |  |  |  |
|  | F1613_RS04220 | UvrB/UvrC motif-containing protein | 5.50 | 3.79E-20 |
|  | F1613_RS03190 | IS1182-like element ISSep1 family transposase | 3.34 | 6.29E-13 |
|  | F1613_RS05970 | IS1182-like element ISSep1 family transposase | 3.27 | 2.26E-11 |
|  | F1613_RS12665 | Transposase | 3.05 | 7.94E-09 |
| dinB | F1613_RS10840 | DNA polymerase IV | 2.53 | 1.31E-11 |
|  | F1613_RS03005 | recombinase family protein | 2.09 | 3.32E-04 |
|  | F1613_RS11430 | single-stranded DNA-binding protein | 1.61 | 2.01E-02 |
|  | F1613_RS10835 | 3'-5' exonuclease | 1.56 | 1.07E-02 |
| **Hypothetical Proteins** |  |  |  |  |
|  | F1613_RS12445 | hypothetical protein | 4.77 | 1.67E-13 |
|  | F1613_RS12480 | hypothetical protein | 4.17 | 1.15E-07 |
|  | F1613_RS09455 | hypothetical protein | 3.59 | 1.99E-05 |
|  | F1613_RS04750 | hypothetical protein | 3.34 | 5.84E-13 |
|  | F1613_RS06495 | hypothetical protein | 2.67 | 1.51E-08 |
|  | F1613_RS04080 | hypothetical protein | 2.40 | 6.26E-05 |
|  | F1613_RS02970 | hypothetical protein | 2.39 | 1.41E-08 |
|  | F1613_RS06155 | hypothetical protein | 2.37 | 1.51E-03 |
|  | F1613_RS01225 | hypothetical protein | 2.34 | 4.15E-06 |
|  | F1613_RS00080 | hypothetical protein | 2.32 | 9.70E-04 |
|  | F1613_RS11815 | hypothetical protein | 2.32 | 1.60E-04 |
|  | F1613_RS12420 | hypothetical protein | 2.32 | 4.15E-04 |
|  | F1613_RS07835 | hypothetical protein | 2.24 | 1.23E-07 |
|  | F1613_RS12725 | hypothetical protein | 2.18 | 2.35E-05 |
|  | F1613_RS10800 | hypothetical protein | 2.12 | 9.64E-05 |
|  | F1613_RS05575 | hypothetical protein | 2.06 | 9.25E-04 |
|  | F1613_RS05560 | hypothetical protein | 2.05 | 1.11E-04 |
|  | F1613_RS12570 | hypothetical protein | 1.98 | 1.18E-02 |
|  | F1613_RS01035 | hypothetical protein | 1.92 | 1.46E-04 |
|  | F1613_RS10225 | hypothetical protein | 1.86 | 7.03E-05 |
|  | F1613_RS07830 | hypothetical protein | 1.77 | 3.99E-05 |
|  | F1613_RS12120 | hypothetical protein | 1.66 | 6.00E-04 |
|  | F1613_RS05215 | hypothetical protein | 1.63 | 1.20E-02 |
| **Metabolism** |  |  |  |  |
| lacG | F1613_RS11900 | 6-phospho-beta-galactosidase | 5.79 | 2.54E-41 |
| lacD | F1613_RS11915 | tagatose-bisphosphate aldolase | 5.79 | 1.38E-26 |
|  | F1613_RS11920 | tagatose-6-phosphate kinase | 5.49 | 2.17E-27 |
| lacB | F1613_RS11925 | galactose-6-phosphate isomerase subunit LacB | 5.20 | 3.64E-25 |
|  | F1613_RS03870 | ArgE/DapE family deacylase | 5.00 | 3.67E-08 |
| lacA | F1613_RS11930 | galactose-6-phosphate isomerase subunit LacA | 4.60 | 1.22E-20 |
|  | F1613_RS01550 | NAD(P)/FAD-dependent oxidoreductase | 4.49 | 5.54E-18 |
|  | F1613_RS01545 | glutathione peroxidase | 4.37 | 9.13E-17 |
| nrdG | F1613_RS01485 | anaerobic ribonucleoside-triphosphate reductase activating protein | 4.30 | 4.32E-20 |
| nrdD | F1613_RS01490 | anaerobic ribonucleoside-triphosphate reductase | 4.23 | 4.82E-20 |
|  | F1613_RS01370 | aspartate aminotransferase family protein | 4.17 | 1.38E-26 |
| ilvC | F1613_RS11225 | ketol-acid reductoisomerase | 3.82 | 5.98E-31 |
|  | F1613_RS03785 | acetoin reductase | 3.72 | 2.26E-12 |
|  | F1613_RS11220 | ACT domain-containing protein | 3.60 | 2.71E-10 |
|  | F1613_RS04060 | thiamine pyrophosphate-dependent dehydrogenase E1 component subunit alpha | 3.55 | 0.003519281 |
|  | F1613_RS04065 | alpha-ketoacid dehydrogenase subunit beta | 3.50 | 0.000297743 |
|  | F1613_RS01135 | zinc-dependent alcohol dehydrogenase family protein | 3.28 | 1.02E-16 |
|  | F1613_RS09310 | LLM class flavin-dependent oxidoreductase | 3.26 | 1.04E-23 |
|  | F1613_RS11230 | 2-isopropylmalate synthase | 3.26 | 3.36E-19 |
|  | F1613_RS06075 | NADH-dependent flavin oxidoreductase | 3.12 | 1.61E-16 |
| leuB | F1613_RS11235 | 3-isopropylmalate dehydrogenase | 3.12 | 7.41E-13 |
| pfkB | F1613_RS05155 | 1-phosphofructokinase | 3.07 | 2.21E-05 |
| gntK | F1613_RS00865 | Gluconokinase | 3.02 | 2.77E-14 |
|  | F1613_RS08045 | aminodeoxychorismate/anthranilate synthase component II | 2.96 | 2.55E-06 |
| leuC | F1613_RS11240 | 3-isopropylmalate dehydratase large subunit | 2.84 | 4.28E-11 |
| pflA | F1613_RS03865 | pyruvate formate-lyase-activating protein | 2.78 | 6.97E-07 |
|  | F1613_RS04620 | FAD-containing oxidoreductase | 2.76 | 1.41E-08 |
|  | F1613_RS09950 | proline dehydrogenase | 2.68 | 0.000151127 |
| ilvB | F1613_RS11215 | biosynthetic-type acetolactate synthase large subunit | 2.66 | 9.89E-12 |
| pruA | F1613_RS01215 | L-glutamate gamma-semialdehyde dehydrogenase | 2.66 | 2.18E-05 |
| adhE | F1613_RS05320 | bifunctional acetaldehyde-CoA/alcohol dehydrogenase | 2.62 | 9.24E-07 |
| alsS | F1613_RS01350 | acetolactate synthase AlsS | 2.61 | 9.89E-12 |
|  | F1613_RS11890 | LLM class flavin-dependent oxidoreductase | 2.52 | 1.32E-12 |
|  | F1613_RS04070 | dihydrolipoamide acetyltransferase family protein | 2.49 | 2.13E-03 |
|  | F1613_RS01000 | aldehyde dehydrogenase family protein | 2.49 | 8.05E-04 |
|  | F1613_RS01355 | L-lactate dehydrogenase | 2.43 | 4.87E-11 |
| leuD | F1613_RS11245 | 3-isopropylmalate dehydratase small subunit | 2.40 | 1.34E-09 |
|  | F1613_RS02610 | PLP-dependent aspartate aminotransferase family protein | 2.39 | 4.74E-09 |
|  | F1613_RS06705 | oleate hydratase | 2.38 | 5.16E-03 |
| budA | F1613_RS01345 | acetolactate decarboxylase | 2.35 | 7.57E-10 |
| ilvA | F1613_RS11250 | threonine ammonia-lyase IlvA | 2.35 | 1.37E-14 |
|  | F1613_RS00685 | 2-dehydropantoate 2-reductase | 2.35 | 2.89E-11 |
| dhaK | F1613_RS03965 | dihydroxyacetone kinase subunit DhaK | 2.32 | 7.57E-09 |
|  | F1613_RS03770 | FAD:protein FMN transferase | 2.29 | 6.60E-08 |
| pflB | F1613_RS03860 | formate C-acetyltransferase | 2.23 | 1.90E-05 |
| dhaL | F1613_RS03970 | dihydroxyacetone kinase subunit DhaL | 2.17 | 3.24E-07 |
| zwf | F1613_RS08725 | glucose-6-phosphate dehydrogenase | 2.14 | 1.27E-08 |
| trpD | F1613_RS08050 | anthranilate phosphoribosyltransferase | 2.10 | 1.12E-06 |
|  | F1613_RS04185 | arylamine N-acetyltransferase | 2.08 | 8.10E-06 |
| ald | F1613_RS09695 | alanine dehydrogenase | 2.07 | 6.95E-06 |
|  | F1613_RS01745 | (S)-acetoin forming diacetyl reductase | 2.07 | 7.75E-05 |
| dhaM | F1613_RS03975 | dihydroxyacetone kinase phosphoryl donor subunit DhaM | 2.04 | 3.29E-06 |
|  | F1613_RS03960 | glycerol dehydrogenase | 2.01 | 3.88E-06 |
| bshC | F1613_RS07090 | bacillithiol biosynthesis cysteine-adding enzyme BshC | 1.96 | 1.39E-08 |
|  | F1613_RS08740 | alpha-glucosidase | 1.95 | 4.58E-07 |
|  | F1613_RS09305 | cysteine desulfurase family protein | 1.94 | 2.52E-06 |
|  | F1613_RS02605 | PLP-dependent transferase | 1.94 | 8.26E-08 |
|  | F1613_RS02260 | glutamate synthase subunit beta | 1.92 | 8.97E-05 |
|  | F1613_RS05795 | thioredoxin family protein | 1.91 | 3.23E-05 |
|  | F1613_RS01010 | NAD(P)H-dependent oxidoreductase | 1.91 | 4.30E-09 |
| arcC | F1613_RS03315 | carbamate kinase | 1.90 | 1.07E-03 |
|  | F1613_RS08040 | anthranilate synthase component I | 1.87 | 1.77E-05 |
|  | F1613_RS03370 | aspartate aminotransferase family protein | 1.85 | 3.49E-10 |
|  | F1613_RS01670 | alpha-keto acid decarboxylase family protein | 1.80 | 1.06E-03 |
|  | F1613_RS00940 | thiazole synthase | 1.75 | 1.22E-04 |
|  | F1613_RS00620 | 2,3-diphosphoglycerate-dependent phosphoglycerate mutase | 1.74 | 1.04E-02 |
| argH | F1613_RS06100 | argininosuccinate lyase | 1.73 | 2.97E-03 |
|  | F1613_RS08195 | alanine racemase | 1.72 | 8.59E-03 |
|  | F1613_RS01635 | poly-gamma-glutamate hydrolase family protein | 1.71 | 1.94E-06 |
| thrB | F1613_RS07855 | homoserine kinase | 1.69 | 2.57E-08 |
| betA | F1613_RS01460 | betaine-aldehyde dehydrogenase | 1.68 | 8.73E-03 |
|  | F1613_RS07840 | aspartate kinase | 1.68 | 1.12E-03 |
| gpmI | F1613_RS05600 | 2,3-bisphosphoglycerate-independent phosphoglycerate mutase | 1.67 | 4.34E-04 |
| thiF | F1613_RS00945 | thiazole biosynthesis adenylyltransferase ThiF | 1.67 | 1.94E-05 |
| thiO | F1613_RS00930 | glycine oxidase ThiO | 1.63 | 2.60E-04 |
| thiS | F1613_RS00935 | sulfur carrier protein ThiS | 1.62 | 2.10E-03 |
|  | F1613_RS02615 | bifunctional homocysteine S-methyltransferase/methylenetetrahydrofolate reductase | 1.62 | 9.07E-05 |
| ilvD | F1613_RS11210 | dihydroxy-acid dehydratase | 1.61 | 4.55E-06 |
| tpiA | F1613_RS05595 | triose-phosphate isomerase | 1.61 | 1.74E-04 |
| nagB | F1613_RS04495 | glucosamine-6-phosphate deaminase | 1.59 | 1.53E-05 |
|  | F1613_RS05590 | phosphoglycerate kinase | 1.58 | 3.64E-04 |
|  | F1613_RS08710 | SDR family NAD(P)-dependent oxidoreductase | 1.58 | 7.92E-04 |
| adhP | F1613_RS04670 | alcohol dehydrogenase AdhP | 1.57 | 2.83E-03 |
| pcp | F1613_RS04095 | pyroglutamyl-peptidase I | 1.54 | 4.71E-05 |
|  | F1613_RS00035 | 2-hydroxyacid dehydrogenase family protein | 1.52 | 4.32E-04 |
| **Molecular Chaperones** |  |  |  |  |
| clpB | F1613_RS06180 | ATP-dependent chaperone ClpB | 6.15 | 6.64E-17 |
|  | F1613_RS04230 | ATP-dependent Clp protease ATP-binding subunit clpC | 5.73 | 4.24E-20 |
| dnaK | F1613_RS09115 | molecular chaperone DnaK | 3.91 | 5.29E-15 |
| grpE | F1613_RS09120 | nucleotide exchange factor GrpE | 3.77 | 8.23E-14 |
| groL | F1613_RS11085 | chaperonin GroEL | 3.51 | 3.99E-17 |
| groES | F1613_RS11090 | co-chaperone GroES | 3.37 | 6.96E-16 |
| dnaJ | F1613_RS09110 | molecular chaperone DnaJ | 2.97 | 2.97E-16 |
| hslO | F1613_RS02040 | Hsp33 family molecular chaperone HslO | 2.05 | 1.68E-04 |
| **Ribosome Biogenesis** |  |  |  |  |
| prmA | F1613_RS09105 | 50S ribosomal protein L11 methyltransferase | 2.95 | 4.32E-14 |
| **Oxidative Phosphorylation** |  |  |  |  |
|  | F1613_RS06745 | cytochrome ubiquinol oxidase subunit I | 4.99 | 2.62E-31 |
|  | F1613_RS06750 | cytochrome d ubiquinol oxidase subunit II | 4.97 | 4.11E-28 |
| **Protein Degradation** |  |  |  |  |
|  | F1613_RS04225 | protein arginine kinase | 5.63 | 9.87E-20 |
| clpP | F1613_RS05555 | ATP-dependent Clp endopeptidase proteolytic subunit ClpP | 3.16 | 1.90E-17 |
| mecA | F1613_RS06250 | adaptor protein MecA | 1.94 | 1.32E-03 |
| yjbH | F1613_RS06265 | protease adaptor protein YjbH | 1.78 | 6.45E-08 |
|  | F1613_RS08220 | CPBP family intramembrane metalloprotease | 1.76 | 1.62E-02 |
|  | F1613_RS09795 | S1C family serine protease | 1.61 | 2.10E-03 |
| **Regulation** |  |  |  |  |
|  | F1613_RS04215 | CtsR family transcription regulator | 5.38 | 5.02E-22 |
|  | F1613_RS01555 | MarR family transcription regulator | 4.90 | 1.29E-23 |
| hrcA | F1613_RS09125 | heat-inducible transcriptional repressor HrcA | 3.83 | 1.35E-15 |
|  | F1613_RS04625 | Rrf2 family transcription regulator | 2.94 | 9.70E-08 |
|  | F1613_RS03175 | metalloregulator ArsR/SmtB family transcription factor | 2.67 | 2.72E-05 |
|  | F1613_RS11940 | NAD-dependent protein deacylase | 2.66 | 2.56E-15 |
|  | F1613_RS01755 | BglG family transcription antiterminator | 2.38 | 2.92E-10 |
|  | F1613_RS00870 | GntR family transcription regulator | 2.37 | 2.86E-07 |
|  | F1613_RS05150 | DeoR/GlpR family DNA-binding transcription regulator | 2.33 | 7.16E-05 |
| saeR | F1613_RS05195 | response regulator transcription factor SaeR | 2.27 | 1.06E-03 |
| raiA | F1613_RS05465 | ribosome-associated translation inhibitor RaiA | 2.26 | 3.45E-05 |
| vraR | F1613_RS10785 | two-component system response regulator VraR | 2.20 | 4.58E-07 |
|  | F1613_RS10000 | competence protein ComK | 2.20 | 1.25E-04 |
|  | F1613_RS09995 | sigma-70 family RNA polymerase sigma factor | 2.17 | 3.48E-04 |
|  | F1613_RS06475 | competence protein ComK | 2.17 | 2.29E-04 |
|  | F1613_RS00070 | HTH domain-containing protein | 2.06 | 1.02E-06 |
|  | F1613_RS02270 | LysR family transcription regulator | 1.99 | 6.00E-07 |
|  | F1613_RS03505 | metalloregulator ArsR/SmtB family transcription factor | 1.79 | 8.05E-05 |
|  | F1613_RS03130 | metalloregulator ArsR/SmtB family transcription factor | 1.61 | 1.52E-03 |
|  | F1613_RS12175 | winged helix DNA-binding protein | 1.57 | 1.66E-02 |
|  | F1613_RS02675 | GNAT family protein | 1.55 | 1.19E-04 |
| **Secretion** |  |  |  |  |
|  | F1613_RS10230 | TrbC/VirB2 family protein | 2.16 | 5.81E-09 |
| asp3 | F1613_RS01845 | accessory Sec system protein Asp3 | 1.75 | 2.19E-07 |
| secA2 | F1613_RS01840 | accessory Sec system translocase SecA2 | 1.52 | 3.51E-06 |
| **Signal Transduction** |  |  |  |  |
|  | F1613_RS05190 | HAMP domain-containing sensor histidine kinase | 2.37 | 8.89E-04 |
|  | F1613_RS10790 | sensor histidine kinase | 2.00 | 1.48E-08 |
| **Stress Response** |  |  |  |  |
| liaF | F1613_RS10795 | cell wall-active antibiotics response protein LiaF | 1.75 | 9.78E-06 |
|  | F1613_RS09700 | universal stress protein | 1.70 | 2.49E-05 |
|  | F1613_RS09680 | universal stress protein | 1.55 | 1.29E-03 |
| **Surface Protein** |  |  |  |  |
|  | F1613_RS06965 | YSIRK-type signal peptide-containing protein | 1.86 | 1.93E-05 |
| **Translation** |  |  |  |  |
|  | F1613_RS10325 | tRNA-Gly | 1.92 | 1.63E-03 |
|  | F1613_RS10535 | tRNA-Cys | 1.51 | 1.64E-02 |
|  | F1613_RS10330 | tRNA-His | 1.50 | 2.63E-02 |
| **Transport** |  |  |  |  |
|  | F1613_RS11905 | lactose-specific PTS transporter subunit EIIC | 5.94 | 2.24E-33 |
|  | F1613_RS11910 | PTS lactose/cellobiose transporter subunit IIA | 5.77 | 8.75E-29 |
|  | F1613_RS03780 | MFS transporter | 4.43 | 4.74E-21 |
|  | F1613_RS03185 | heavy metal translocating P-type ATPase | 3.79 | 1.50E-07 |
|  | F1613_RS05160 | PTS fructose transporter subunit IIABC | 3.72 | 3.85E-06 |
|  | F1613_RS06755 | TrkA family potassium uptake protein | 3.11 | 6.97E-16 |
|  | F1613_RS01230 | heavy metal translocating P-type ATPase | 3.05 | 8.08E-11 |
| cydC | F1613_RS05080 | thiol reductant ABC exporter subunit CydC | 3.02 | 3.85E-15 |
|  | F1613_RS05075 | ABC transporter ATP-binding protein/permease | 2.85 | 8.17E-15 |
|  | F1613_RS08655 | ECF transporter S component | 2.63 | 4.33E-09 |
|  | F1613_RS03925 | YfcC family protein | 2.27 | 1.12E-06 |
|  | F1613_RS03435 | SLC45 family MFS transporter | 2.27 | 1.05E-10 |
|  | F1613_RS04815 | sodium:proton antiporter | 2.20 | 3.02E-13 |
|  | F1613_RS02280 | YibE/F family protein | 2.14 | 5.84E-11 |
|  | F1613_RS03180 | ZIP family metal transporter | 2.13 | 4.26E-04 |
|  | F1613_RS08210 | ATP-binding cassette domain-containing protein | 1.88 | 1.35E-03 |
|  | F1613_RS08205 | ABC transporter permease | 1.72 | 6.96E-03 |
|  | F1613_RS10695 | ABC transporter ATP-binding protein | 1.71 | 1.73E-04 |
|  | F1613_RS03910 | osmoprotectant ABC transporter substrate-binding protein | 1.70 | 1.31E-06 |
| arsA | F1613_RS03495 | arsenical pump-driving ATPase | 1.68 | 7.17E-05 |
|  | F1613_RS03915 | ABC transporter permease | 1.68 | 4.39E-06 |
| yut | F1613_RS12295 | urea transporter | 1.64 | 1.23E-03 |
| arsD | F1613_RS03500 | arsenite efflux transporter metallochaperone ArsD | 1.62 | 3.60E-04 |
| rarD | F1613_RS04040 | EamA family transporter RarD | 1.61 | 2.10E-03 |
|  | F1613_RS02275 | YibE/F family protein | 1.56 | 3.52E-03 |
|  | F1613_RS10095 | Ltp family lipoprotein | 1.56 | 1.53E-03 |
|  | F1613_RS05000 | globin domain-containing protein | 1.52 | 2.11E-03 |
|  | F1613_RS04755 | DMT family transporter | 1.52 | 4.03E-04 |
|  | F1613_RS06820 | ABC transporter permease | 1.52 | 2.48E-04 |
|  | F1613_RS00665 | amino acid permease | 1.51 | 3.13E-05 |
|  | F1613_RS04030 | ABC-ATPase domain-containing protein | 1.51 | 4.08E-05 |
| **tRNA Biosynthesis** |  |  |  |  |
| mnmA | F1613_RS09300 | tRNA 2-thiouridine(34) synthase MnmA | 1.54 | 1.89E-03 |
| **Unknown Function** |  |  |  |  |
|  | F1613_RS04110 | YceI family protein | 4.01 | 1.32E-19 |
|  | F1613_RS12660 | uncharacterized gene | 3.91 | 6.02E-21 |
|  | F1613_RS11080 | cell wall surface anchor protein | 3.79 | 3.50E-17 |
|  | F1613_RS07270 | TM2 domain-containing protein | 3.19 | 3.85E-06 |
|  | F1613_RS08215 | Msa family membrane protein | 3.06 | 0.000107032 |
|  | F1613_RS02870 | alpha/beta fold hydrolase | 2.66 | 3.51E-05 |
|  | F1613_RS12185 | uncharacterized gene | 2.62 | 0.001381984 |
|  | F1613_RS12630 | uncharacterized gene | 2.37 | 3.88E-06 |
|  | F1613_RS12510 | uncharacterized gene | 2.35 | 1.36E-05 |
|  | F1613_RS05200 | DoxX family protein | 2.28 | 1.39E-03 |
|  | F1613_RS04970 | Bax inhibitor-1 family protein | 2.27 | 1.19E-07 |
|  | F1613_RS05565 | TIGR01777 family oxidoreductase | 2.20 | 1.27E-08 |
|  | F1613_RS07920 | DUF896 domain-containing protein | 2.18 | 9.44E-08 |
|  | F1613_RS05205 | DM13 domain-containing protein | 2.17 | 2.97E-03 |
|  | F1613_RS03145 | uncharacterized gene | 1.99 | 2.82E-03 |
|  | F1613_RS12190 | uncharacterized gene | 1.95 | 1.66E-05 |
|  | F1613_RS09745 | GAF domain-containing protein | 1.78 | 1.96E-06 |
|  | F1613_RS12520 | uncharacterized gene | 1.77 | 1.74E-03 |
|  | F1613_RS01120 | alpha/beta hydrolase | 1.66 | 3.89E-04 |
| yidD | F1613_RS10075 | membrane protein insertion efficiency factor YidD | 1.61 | 1.90E-04 |
|  | F1613_RS06170 | metal-sulfur cluster assembly factor | 1.58 | 4.71E-05 |
|  | F1613_RS03920 | alpha/beta hydrolase family protein | 1.54 | 4.87E-05 |
|  | F1613_RS03440 | uncharacterized gene | 1.54 | 1.55E-03 |
|  | F1613_RS12555 | uncharacterized gene | 1.51 | 3.13E-04 |
| **Urease Accessory Proteins** |  |  |  |  |
|  | F1613_RS12320 | urease accessory protein UreF | 1.59 | 3.99E-03 |
| ureE | F1613_RS12315 | urease accessory protein UreE | 1.52 | 5.22E-03 |
|  | F1613_RS12330 | urease accessory protein UreD | 1.50 | 4.91E-03 |
| **Virulence Factors** |  |  |  |  |
| vraX | F1613_RS04535 | C1q-binding complement inhibitor VraX | 5.42 | 3.72E-10 |
| blaZ | F1613_RS10980 | penicillin-hydrolyzing class A beta-lactamase BlaZ | 4.19 | 9.08E-13 |
| blaI | F1613_RS10970 | penicillinase repressor BlaI | 2.32 | 5.68E-08 |
| blaR1 | F1613_RS10975 | beta-lactam sensor/signal transducer BlaR1 | 2.25 | 3.96E-06 |
