# Supplementary material for: Genome-wide transcription response of Staphylococcus epidermidis to heat shock and medically relevant glucose levels
Source: Front Microbiol. 2024 Jul 22;15:1408796. doi: 10.3389/fmicb.2024.1408796 (PMC11298487; doi:10.3389/fmicb.2024.1408796)
Supplement: Supplementary file 8 [file Table_2.DOCX]

**Table S2| *S. epidermidis* heat-shock repressed transcripts.**

| **Functional Class**  **& Symbol** | **Locus** | **Name** | **log2FC** | **Adjusted p-value** |
| --- | --- | --- | --- | --- |
| **Beta-Class Phenol-Soluble Modulin** |  |  |  |  |
|  | F1613_RS07075 | beta-class phenol-soluble modulin | -2.20 | 2.87E-03 |
|  | F1613_RS07060 | beta-class phenol-soluble modulin | -1.89 | 8.67E-03 |
|  | F1613_RS07065 | beta-class phenol-soluble modulin | -1.63 | 1.90E-02 |
|  | F1613_RS07070 | beta-class phenol-soluble modulin | -1.55 | 2.47E-02 |
| **Cell Wall Structure** |  |  |  |  |
|  | F1613_RS05940 | teichoic acid D-Ala incorporation-associated protein DltX | -4.40 | 1.27E-08 |
| dltA | F1613_RS05945 | D-alanine--poly(phosphoribitol) ligase subunit DltA | -3.73 | 1.04E-05 |
| dltB | F1613_RS05950 | D-alanyl-lipoteichoic acid biosynthesis protein DltB | -3.63 | 2.18E-05 |
| dltC | F1613_RS05955 | D-alanine--poly(phosphoribitol) ligase subunit 2 | -3.38 | 5.78E-05 |
| dltD | F1613_RS05960 | D-alanyl-lipoteichoic acid biosynthesis protein DltD | -3.34 | 6.11E-06 |
|  | F1613_RS01265 | transglycosylase family protein | -3.08 | 2.98E-05 |
| **DNA Restriction-Modification System** |  |  |  |  |
|  | F1613_RS03040 | restriction endonuclease subunit R | -1.53 | 3.87E-02 |
| **DNA/RNA Repair** |  |  |  |  |
| ssb | F1613_RS02565 | single-stranded DNA-binding protein | -3.06 | 1.81E-11 |
|  | F1613_RS09000 | deoxyribonuclease IV | -1.66 | 6.76E-03 |
| radC | F1613_RS09460 | DNA repair protein RadC | -1.59 | 4.52E-03 |
| **Hypothetical Protein** |  |  |  |  |
|  | F1613_RS00595 | hypothetical protein | -2.90 | 7.05E-13 |
|  | F1613_RS10130 | hypothetical protein | -2.60 | 2.50E-03 |
|  | F1613_RS03300 | hypothetical protein | -2.55 | 2.06E-09 |
|  | F1613_RS05655 | hypothetical protein | -2.27 | 7.58E-12 |
|  | F1613_RS07970 | hypothetical protein | -2.23 | 3.40E-06 |
|  | F1613_RS01415 | hypothetical protein | -2.14 | 2.15E-06 |
|  | F1613_RS04530 | hypothetical protein | -1.98 | 1.85E-03 |
|  | F1613_RS05640 | hypothetical protein | -1.95 | 2.57E-07 |
|  | F1613_RS10300 | hypothetical protein | -1.82 | 2.69E-02 |
|  | F1613_RS10270 | hypothetical protein | -1.80 | 6.56E-03 |
|  | F1613_RS05665 | hypothetical protein | -1.79 | 2.48E-03 |
|  | F1613_RS02945 | hypothetical protein | -1.78 | 5.50E-06 |
|  | F1613_RS12485 | hypothetical protein | -1.77 | 6.93E-05 |
|  | F1613_RS12415 | hypothetical protein | -1.71 | 6.67E-10 |
|  | F1613_RS00295 | hypothetical protein | -1.63 | 1.55E-03 |
|  | F1613_RS11670 | hypothetical protein | -1.58 | 1.08E-06 |
| **Metabolism** |  |  |  |  |
|  | F1613_RS03290 | NADP-dependent oxidoreductase | -3.35 | 2.30E-15 |
|  | F1613_RS10115 | Amidase | -2.80 | 7.12E-05 |
|  | F1613_RS11355 | DEAD/DEAH box helicase | -2.74 | 2.11E-06 |
|  | F1613_RS07205 | aspartate carbamoyltransferase catalytic subunit | -2.56 | 4.68E-08 |
|  | F1613_RS00215 | galactose mutarotase | -2.40 | 2.29E-10 |
|  | F1613_RS01150 | pyruvate oxidase | -2.18 | 5.95E-04 |
| plsY | F1613_RS07975 | glycerol-3-phosphate 1-O-acyltransferase PlsY | -2.17 | 1.83E-10 |
|  | F1613_RS04320 | class I SAM-dependent methyltransferase | -2.13 | 5.08E-04 |
|  | F1613_RS04425 | deoxynucleoside kinase | -2.00 | 1.50E-05 |
|  | F1613_RS04420 | deoxynucleoside kinase | -1.99 | 2.73E-05 |
|  | F1613_RS11585 | CTP synthase | -1.97 | 6.08E-11 |
|  | F1613_RS07080 | YjjG family noncanonical pyrimidine nucleotidase | -1.93 | 6.33E-05 |
| rpe | F1613_RS07310 | ribulose-phosphate 3-epimerase | -1.84 | 5.08E-04 |
|  | F1613_RS09005 | DEAD/DEAH box helicase | -1.82 | 3.48E-03 |
|  | F1613_RS07500 | isoprenyl transferase | -1.57 | 4.14E-04 |
|  | F1613_RS04520 | acetyl-CoA C-acyltransferase | -1.57 | 2.21E-05 |
| galU | F1613_RS00855 | UTP--glucose-1-phosphate uridylyltransferase GalU | -1.56 | 7.96E-05 |
| rnmV | F1613_RS02140 | ribonuclease M5 | -1.54 | 8.77E-03 |
| coaW | F1613_RS11600 | type II pantothenate kinase | -1.54 | 8.24E-06 |
|  | F1613_RS07215 | carbamoyl phosphate synthase small subunit | -1.51 | 6.19E-05 |
| **Metal-Binding Proteins** |  |  |  |  |
|  | F1613_RS09010 | Nif3-like dinuclear metal center hexameric protein | -1.81 | 3.82E-04 |
| **DNA Metabolic Process** |  |  |  |  |
|  | F1613_RS05645 | tyrosine-type recombinase/integrase | -2.69 | 2.39E-05 |
| **Regulation** |  |  |  |  |
|  | F1613_RS10440 | helix-turn-helix transcription regulator | -3.08 | 1.57E-12 |
| rsp | F1613_RS00450 | AraC family transcription regulator Rsp | -2.47 | 2.96E-05 |
|  | F1613_RS11065 | GntR family transcription regulator | -2.20 | 5.03E-04 |
| pyrR | F1613_RS07195 | bifunctional pyr operon transcription regulator/uracil phosphoribosyltransferase PyrR | -2.04 | 5.00E-03 |
|  | F1613_RS09035 | helix-turn-helix transcription regulator | -1.81 | 1.58E-03 |
| **Ribosome Biogenesis** |  |  |  |  |
| rsgA | F1613_RS07305 | ribosome small subunit-dependent GTPase A | -1.73 | 3.23E-07 |
| **Stress Response** |  |  |  |  |
|  | F1613_RS05710 | cold-shock protein | -3.18 | 1.16E-08 |
| cspA | F1613_RS08225 | cold shock protein CspA | -2.71 | 2.21E-05 |
|  | F1613_RS02545 | GlsB/YeaQ/YmgE family stress response membrane protein | -1.96 | 1.30E-03 |
| **Transcription Machinery** |  |  |  |  |
| rpoE | F1613_RS11590 | DNA-directed RNA polymerase subunit delta | -1.94 | 8.91E-07 |
| **Ribosome/ Translation** |  |  |  |  |
| rpsF | F1613_RS02570 | 30S ribosomal protein S6 | -3.05 | 2.58E-10 |
|  | F1613_RS01980 | *tRNA-Arg | -3.01 | 3.86E-07 |
| rpsR | F1613_RS02560 | 30S ribosomal protein S18 | -2.82 | 9.62E-06 |
|  | F1613_RS11735 | tRNA-Lys | -2.62 | 1.61E-04 |
| typA | F1613_RS06860 | translational GTPase TypA | -2.49 | 1.08E-06 |
|  | F1613_RS01985 | *tRNA-Leu | -2.47 | 1.28E-05 |
| infC | F1613_RS09555 | translation initiation factor IF-3 | -2.27 | 2.13E-08 |
| rpmI | F1613_RS09550 | 50S ribosomal protein L35 | -2.26 | 8.10E-06 |
| rplJ | F1613_RS04310 | 50S ribosomal protein L10 | -2.24 | 1.64E-04 |
|  | F1613_RS09040 | glycine--tRNA ligase | -2.18 | 9.70E-05 |
| rplT | F1613_RS09545 | 50S ribosomal protein L20 | -2.17 | 4.26E-04 |
| rpsO | F1613_RS07565 | 30S ribosomal protein S15 | -2.16 | 1.26E-04 |
| rplL | F1613_RS04315 | 50S ribosomal protein L7/L12 | -2.02 | 1.31E-03 |
|  | F1613_RS10455 | RluA family pseudouridine synthase | -2.02 | 1.61E-08 |
| ileS | F1613_RS07170 | isoleucine--tRNA ligase | -1.76 | 2.15E-05 |
| thrS | F1613_RS09565 | threonine--tRNA ligase | -1.65 | 6.30E-03 |
| serS | F1613_RS02850 | serine--tRNA ligase | -1.51 | 1.15E-03 |
| **Transport** |  |  |  |  |
|  | F1613_RS03285 | ABC transporter permease | -3.47 | 6.35E-17 |
|  | F1613_RS03280 | ABC transporter ATP-binding protein | -3.33 | 7.19E-13 |
|  | F1613_RS07200 | NCS2 family nucleobase:cation symporter | -3.23 | 5.01E-05 |
|  | F1613_RS00310 | HlyD family efflux transporter periplasmic adaptor subunit | -2.87 | 6.45E-08 |
| ptsG | F1613_RS01145 | glucose-specific PTS transporter subunit IIBC | -2.77 | 1.44E-06 |
|  | F1613_RS00745 | amino acid permease | -2.55 | 2.83E-06 |
|  | F1613_RS00305 | DHA2 family efflux MFS transporter permease subunit | -2.46 | 5.64E-06 |
|  | F1613_RS05370 | ABC transporter permease | -2.22 | 8.18E-04 |
|  | F1613_RS08160 | ATP-binding cassette domain-containing protein | -2.22 | 4.12E-08 |
| pmtA | F1613_RS11060 | phenol-soluble modulin export ABC transporter ATP-binding protein PmtA | -2.12 | 4.32E-04 |
|  | F1613_RS03990 | MDR family MFS transporter | -2.06 | 4.17E-05 |
|  | F1613_RS05375 | iron chelate uptake ABC transporter family permease subunit | -2.03 | 5.52E-04 |
|  | F1613_RS05380 | ATP-binding cassette domain-containing protein | -2.02 | 4.98E-04 |
|  | F1613_RS10505 | PTS transporter subunit IIC | -2.01 | 1.25E-10 |
| pmtB | F1613_RS11055 | phenol-soluble modulin export ABC transporter permease subunit PmtB | -2.01 | 6.48E-04 |
|  | F1613_RS05385 | siderophore ABC transporter substrate-binding protein | -1.90 | 8.92E-04 |
|  | F1613_RS11970 | energy-coupling factor transporter ATPase | -1.81 | 5.01E-05 |
|  | F1613_RS11840 | Fe(3+) dicitrate ABC transporter substrate-binding protein | -1.80 | 6.41E-03 |
|  | F1613_RS09635 | amino acid permease | -1.78 | 1.83E-05 |
|  | F1613_RS04525 | protein VraC | -1.76 | 3.81E-03 |
| pmtC | F1613_RS11050 | phenol-soluble modulin export ABC transporter ATP-binding protein PmtC | -1.68 | 5.58E-03 |
|  | F1613_RS02750 | RND transporter | -1.65 | 1.37E-02 |
|  | F1613_RS00160 | Na+/H+ antiporter NhaC family protein | -1.54 | 9.05E-06 |
| mscL | F1613_RS07950 | large conductance mechanosensitive channel protein MscL | -1.55 | 6.45E-08 |
| **tRNA Biosynthesis** |  |  |  |  |
|  | F1613_RS09015 | tRNA (adenine(22)-N(1))-methyltransferase TrmK | -1.94 | 5.14E-05 |
| rsmA | F1613_RS02135 | 16S rRNA (adenine(1518)-N(6)/adenine(1519)-N(6))-dimethyltransferase RsmA | -1.69 | 6.43E-04 |
|  | F1613_RS01310 | epoxyqueuosine reductase QueH | -1.62 | 3.53E-03 |
|  | F1613_RS02165 | tRNA1(Val) (adenine(37)-N6)-methyltransferase | -1.56 | 3.51E-04 |
| **Unknown Function** |  |  |  |  |
|  | F1613_RS03295 | HXXEE domain-containing protein | -3.20 | 3.60E-16 |
|  | F1613_RS10435 | DUF445 family protein | -2.78 | 5.43E-09 |
|  | F1613_RS10135 | AAA family ATPase | -2.78 | 6.32E-04 |
|  | F1613_RS02640 | sterile alpha motif-like domain-containing protein | -2.36 | 3.52E-03 |
|  | F1613_RS11185 | AAA family ATPase | -2.20 | 1.19E-04 |
|  | F1613_RS01255 | CHAP domain-containing protein | -2.16 | 1.81E-03 |
|  | F1613_RS01155 | LrgB family protein | -2.14 | 2.61E-04 |
|  | F1613_RS05980 | YuzD family protein | -2.11 | 5.16E-03 |
|  | F1613_RS02400 | VOC family protein | -2.03 | 4.23E-04 |
|  | F1613_RS04965 | LysM peptidoglycan-binding domain-containing protein | -2.02 | 5.53E-05 |
|  | F1613_RS09160 | helix-hairpin-helix domain-containing protein | -1.99 | 1.06E-11 |
|  | F1613_RS12355 | CHAP domain-containing protein | -1.95 | 5.56E-03 |
|  | F1613_RS05400 | GrpB family protein | -1.94 | 8.77E-03 |
|  | F1613_RS03270 | HXXEE domain-containing protein | -1.88 | 7.30E-04 |
|  | F1613_RS04510 | HAD family hydrolase | -1.86 | 1.34E-02 |
|  | F1613_RS00015 | DUF4870 domain-containing protein | -1.83 | 2.60E-08 |
|  | F1613_RS00590 | Aminoacyltransferase | -1.81 | 7.80E-07 |
|  | F1613_RS04515 | uncharacterized gene | -1.78 | 4.23E-04 |
|  | F1613_RS06565 | acyltransferase family protein | -1.75 | 7.43E-08 |
|  | F1613_RS07820 | membrane protein | -1.60 | 5.04E-03 |
|  | F1613_RS12655 | uncharacterized gene | -1.56 | 2.90E-02 |
|  | F1613_RS01730 | DUF1672 domain-containing protein | -1.52 | 5.96E-03 |
|  | F1613_RS04915 | membrane protein | -1.51 | 3.01E-09 |
| **Virulence Factors** |  |  |  |  |
| mprF | F1613_RS08005 | bifunctional lysylphosphatidylglycerol flippase/synthetase MprF | -1.74 | 3.71E-03 |
